# International Paralympic Committee (IPC) and International Blind Sports Federation (IBSA) Joint Position Stand on the Sport-Specific Classification of Athletes with Vision Impairment

**DOI:** 10.1007/s40279-018-0949-6

**Published:** 2018-07-09

**Authors:** David L. Mann, H. J. C. Ravensbergen

**Affiliations:** Department of Human Movement Sciences, Amsterdam Movement Sciences and Institute of Brain and Behavior Amsterdam, Vrije Universiteit Amsterdam, Van der Boechorststraat 9, 1081 BT Amsterdam, The Netherlands

## Abstract

Classification is a defining characteristic of para-sports whereby eligible athletes are allocated a sport class to compete against others with similar activity limitations. To account for the unique characteristics of each sport, para-sports should develop their own classification system using evidence that demonstrates the impact of impairment on performance in that sport. Although the move towards sport-specific classification has progressed in sports for athletes with physical and intellectual impairments, sports for athletes with vision impairment (VI) continue to use the same three classes irrespective of the sport, with classes delineated by legal definitions of low vision and blindness. The aim of this joint International Paralympic Committee/International Blind Sports Federation (IPC/IBSA) Position Stand is to provide guidance for how evidence-based sport-specific classification should be achieved in VI sports. It does so by outlining three conceptual research models (correlation, simulation, and component analysis) that can be used to establish both the minimum impairment required to compete plus the appropriate number of sport classes and their inclusion criteria. The present evaluation of vision relies on measures of visual acuity and field, but new criteria may require a sport-specific combination of additional measures of visual function (e.g. contrast, motion, and light sensitivity) to better account for the impact of VI on sport performance. Moreover, the test procedures used during athlete evaluation (e.g. whether to evaluate both eyes individually or together) should be chosen to better represent the habitual viewing situation experienced in that sport. The development of sport-specific criteria should enhance the legitimacy of competition and encourage increased grassroots participation in VI sports.

## Key Points

**Table:** 

Three research models are described for establishing sport-specific classification criteria for the minimum level of impairment, and sport classes, for athletes with vision impairment (VI).
Sports should investigate whether the use of additional measures of visual function (e.g. contrast and motion sensitivity) would improve the evaluation of VI.
The test procedures used when evaluating an athlete’s vision should represent the habitual conditions experienced in the sport.

## Introduction

Para-sports provide tremendous opportunities to enhance the physical, social, and psychological well-being of people with impairment [1–3]. In providing these opportunities, an important goal of para-sport is that the winner of competition should be the best athlete, rather than the athlete with the least impairment. To achieve this goal, sport classes are created so that athletes with impairment are grouped to compete against others who possess a similar level of activity limitation. The process of allocating athletes to a sport class is termed classification. The explicit aim of classification is to minimise the impact of impairment on the outcome of competition [4].

Historically, classification for para-athletes has been performed on the basis of the medical diagnosis of the athlete’s impairment (e.g. level of amputation or spinal cord injury), without necessarily considering the impact of the impairment on performance in that athlete’s sport. This has meant that each athlete’s class might be the same across different sports, despite what were sometimes very obvious differences in the requirements of those sports. As a result, it has been recognised that classification should take into account the specific impact of impairment on performance in a given sport. For instance, an athlete with an amputated hand will possess a significant activity limitation when rowing, and should therefore qualify to compete in para-rowing; however, the amputation is unlikely to limit performance in long-distance running, therefore the athlete should not qualify to compete in para-running. Similarly, in sports for athletes with vision impairment (VI sports), a mild impairment to the central field of vision could result in a significant activity limitation in sports such as shooting or archery, but not necessarily in other sports such as swimming or running. In that case, an athlete with mild central vision loss should qualify to compete in para-shooting and para-archery, but not para-swimming or para-running. Accordingly, a sport-specific approach is required when evaluating the relationship between impairment and sport performance.

The *IPC Athlete Classification Code* [5], first published as the *IPC Classification Code* in 2007, formally introduced the requirement for all para-sports to develop their own sport-specific system of classification. Specifically, sports were required to adopt a classification system formed on the basis of evidence which demonstrates the impact of impairment on performance in that sport. To assist with this process, in 2011 the International Paralympic Committee (IPC) endorsed a Position Stand [4] that articulates guidelines for how an evidence-based system of classification can be developed. However, while publication of the 2011 Position Stand underpinned a move towards sport-specific classification in sports that cater for athletes with physical and intellectual impairments, the progress has been much slower in sports for athletes with VI. Although the 2011 Position Stand was written to be applicable to all para-sports, it was developed largely on the basis of experience in classification for athletes with physical impairment. Some of the unique characteristics of VI, and the unique adaptations made to VI sports, including the use of blindfolds and guides, has led to the need for clarity about how the principles from the 2011 Position Stand would apply to VI sports. Therefore, the aim of this new joint Position Stand is to provide guidance for how evidence-based classification should be achieved in VI sports. This current paper adopts, endorses, and expands on the principles of the 2011 Position Stand, to address pertinent issues that are largely unique for athletes with VI. The paper does so by outlining (i) how evidence-based classification should account for the adaptations commonly used in VI sports; (ii) how the athlete evaluation should be conducted during classification; and (iii) the research approaches that can be adopted to establish an evidence-based system of classification in VI sports.

## The Impact of Sport Rules on Vision Impairment (VI) Classification

Most sports within the Paralympic movement are not identical to their able-bodied equivalent, but instead adopt different sport and equipment rules to better account for the capabilities of people with impairment. For instance, in the regular unadapted form of judo played by those without impairment, athletes start the bout by fighting to obtain the most advantageous grip of their opponent, before continuing to compete with the grip in place. However, in the adapted form of judo played by those with VI, athletes start with the grip already in place, so that the athlete with better vision does not hold a substantial advantage in obtaining a grip on their opponent. A variety of sport adaptations of this type are used within VI sports, and those adaptations hold significant consequences for the way in which research into evidence-based classification should be conducted.

There are two important steps involved in classification for an athlete who wishes to take part in para-sport. In the first step, the athlete must have an eligible impairment that meets the minimum impairment criterion (MIC), defined as “the level of impairment that has an impact on sport performance” [6, 7]. Crucially, the MIC for para-sports should be the level of impairment that decreases an athlete’s level of performance in the unadapted form of the sport. That is, if a judoka’s VI impacts their ability to compete when following the rules of the unadapted form of the sport (i.e., when required to obtain a grip on their opponent), then they should be eligible to take part in the para-version of the sport. The second step during classification requires an athlete to be allocated a sport class so he/she competes against others with a similar activity limitation. Because para-athletes will compete against each other in the adapted form of the sport, the sport class should be determined on the basis of the impact of the impairment on performance in the adapted form of the sport. If two judokas with VI have different levels of impairment that provide one with an advantage when obtaining a grip, yet no advantage when the grip is in place, then the two athletes should compete in the same sport class.

The decision to make adaptations to sport rules and equipment in VI sports is the responsibility of the individual sport federations responsible for governing those sports (for a list of those federations, see Tweedy et al. [8]). Federations generally try to minimise these adaptations so that the sport remains as similar as possible to the sighted version; however, it should now be clear that, when made, these modifications hold significant implications for evidence-based classification. In turn, the outcomes of classification research can lead Federations to consider changes to their technical rules. Below we address two modifications commonly used in VI sport (blindfolds and guides) that have significant implications for how evidence-based classification should be established.

### Blindfolds

Blindfolds (or eyeshades) are used in some VI sports to ensure that all athletes have an equivalent level of impairment during competition (i.e. full blindness). Some VI sports require all athletes to wear a blindfold (e.g. goalball), while other sports require only a subset of athletes to do so (e.g. those with the most severe impairment, as is presently the case for VI swimming).

It has been suggested on occasion that a suitable approach to minimise the impact of impairment on the outcome of competition in VI sport would be to require all athletes to wear a blindfold during competition irrespective of the severity of the athlete’s VI. However, there are several reasons why most people in VI sports do not accept this view [9]. First, a blindfold will add to the existing VI of an athlete who is not completely blind, limiting their ability to capitalise on their remaining vision, a skill that is developed through training. Second, there is concern that when compared with those who are completely blind, those who have some degree of vision but compete with a blindfold might in certain cases perform better during competition because they benefit from having vision away from competition. Indeed, those with some vision may have an advantage not only in their ability to access training but also during training; for instance, when visually modelling their actions on others and when using visual feedback (e.g. video). Conversely, those who are completely blind could, in some cases, have an advantage if competing with a blindfold because they may be better adapted to living and competing when fully blind. Evidently, a consultation process with experts in VI sports revealed that rather than being required to use blindfolds, most VI athletes would prefer to compete without one, even if that meant that they might need to compete in a class against others who have less impairment than they do [9].

Although the use of blindfolds in VI sports remains a controversial issue, there is general agreement that blindfolds are acceptable in some situations (for a discussion see Ravensbergen et al. [9]). The decision to use blindfolds within a given sport is at the discretion of the individual sport federation governing that sport, although the decision does hold significant implications for how a system of classification should be developed. Given that the minimum impairment criteria should be established on the basis of performance in the unadapted form of a sport, then the criteria should be established by examining performance without the blindfold in place. However, the nature of the research to be performed to establish the sport classes will depend on whether either some or all athletes are required to wear a blindfold during competition.

If all athletes are required to wear a blindfold during competition, then it can be argued that only one class should be necessary because all athletes will have the same level of VI during competition. Further research is unlikely to be necessary because there is no need to establish the relationship between impairment and performance when all athletes have the same level of impairment. If research was to be conducted with the blindfolds in place, and did find better performance by those who have less impairment, then this would imply that differences away from competition (e.g. advantages during training), or perhaps the development of other skills (e.g. balance), have a direct impact on performance during competition. Typically, these factors should not be accounted for during classification.

If only a subset of athletes is required to use blindfolds during competition, then research for the allocation of sport classes should be performed on the basis of performance when those athletes required to wear a blindfold do have the blindfold in place. This can present a particular challenge for evidence-based classification because it will not be clear what an athlete’s level of performance might be like without the blindfold in place. When only some athletes must wear a blindfold, it is typically only those who compete in the class for athletes with the most severe VI who must do so. Conceptually, an athlete should be placed into this class when they (1) would be disadvantaged when competing, without the blindfold, against others with less impairment, and (2) would not have an advantage when competing, either with or without the blindfold, against others with more impairment. Consider, for example, VI swimming, where athletes with the most impairment, who currently compete in class ‘S11’, must swim with fully blackened goggles (unless they have two prosthetic eyes), while those with less impairment in classes ‘S13’ and ‘S12’ do not use blackened goggles. Within class S11, most athletes are blind, but there is also a subset of athletes who have a minimal level of vision. Those athletes typically only have the ability to perceive light, yet must compete blindfolded, presumably at least in part to remove any perceived advantage they might have over others who are fully blind. At present though it remains unclear whether those athletes who can perceive light would possess any advantage if allowed to compete without the blindfold. Research is required in that case to show whether the athletes who have minimal vision would perform better without the blackened goggles. Accordingly, an evidence-based decision can be made about whether those athletes should compete in a different class to those who are fully blind, either in a class against athletes with less impairment, or in a separate class altogether. If so, the sport federation should reconsider their requirement for those athletes to compete blindfolded.

### Guides

People with VI frequently experience difficulties in their ability to move safely through their environment. A number of sports such as triathlon and skiing account for this impairment in orientation and mobility [10] by allowing VI athletes to be accompanied by a guide, whose function is to safely lead the athlete during competition. Whereas blindfolds can generally limit or diminish an athlete’s ability to perform, guides help to optimise an athlete’s performance.

The choice to allow guides during competition is another rule-related issue that is at the discretion of a sport federation. If a guide is used in the adapted form of the sport, then the minimum impairment criteria should once again be established on the basis of performance without the guide when competing in the unadapted form of the sport, while the determination of sport classes should be done when considering performance with the guide. However, in some sports, the overall performance of an athlete will depend, at least in part, on the individual contribution of the guide, and therefore this ideally needs to be controlled when establishing the relationship between impairment and performance during classification research. For example, in VI para-cycling, athletes compete while riding a tandem bike with their guide, with both the athlete and their guide cycling the pedals. Therefore, the athlete’s overall performance will depend not only on their own contribution but also that of the guide. For the purposes of classification research, the contribution of the guide should be controlled or eliminated when establishing the impact of impairment on an athlete’s performance, for instance, by standardising the contribution of the guide (e.g. using the same guide for all athletes); limiting the contribution of the guide to one of orientation rather than also contributing to locomotion (unless required for safety); or testing the performance of the athlete without the guide present, using modified equipment (e.g. a stationary bicycle ergometer) while simulating competition.

## Procedure for the Evaluation of Vision During Classification

We now turn to consideration of how the principles of sport-specific classification should also apply to the procedures used when evaluating the vision of athletes during classification, and the implications for classification research. Historically, the criteria used for the sport classes in VI sport have been based largely on the medical definitions of low vision and blindness adopted by the World Health Organization [11]. As a result, the tests performed during classification, and the conditions in which they are tested, have largely been medical. For instance, classification is presently conducted using clinical tests of visual acuity and visual fields, with a minimum level of impairment required on at least one of those tests (≥ 1.0 logMAR or ≤ 20° radius, respectively) for an athlete to be eligible to compete in VI sports. However, the relationship between performance on those tests and sport performance remains largely unclear. Consistent with the principles outlined in the 2011 Position Stand, changes to the way that vision is tested during classification are likely to be required to better capture the way that VI impacts sport performance. Here we address procedural factors for vision testing (e.g. lighting, and testing of the best, or both, eyes) that need to be taken into account to better achieve this aim during athlete evaluation.

### Generic Versus Sport-Specific Tests

Vision tests that are suitable for classification should adequately measure a specific impairment type and should be resistant to improvement as a result of training [4]. If a well-trained athlete was to improve his/her vision test performance as a result of training yet there was no change in the underlying impairment, then the athlete’s class would incorrectly change. The test results would make it appear as though the athlete’s impairment had decreased, and could place the athlete in a class that required them to compete against others with less impairment. The need for tests of vision to be resistant to training holds important implications for the tests that are most likely to be suitable for the purposes of VI classification.

Colenbrander [12] makes a distinction between tests of visual function that measure the capacity of the visual system to ‘see’, and tests of functional vision that assess how a person performs on everyday vision-related tasks. While tests of visual function are those that are typically adopted in medical/clinical settings, tests of functional vision are relevant when evaluating the ability of a person to perform activities of daily living. For example, the test of visual acuity is the most common test of visual function, used to evaluate the ability to read letters on a letter chart at a distance of 4–6 m. However, performance on a test of visual acuity might not necessarily predict how well a person performs when reading a book or driving a car [13]. Instead, respective tests of functional vision (e.g. reading speed or driving performance) are more likely to capture the impact of impairment on task performance. When applied, for example, to swimming, a test that would evaluate an athlete’s ability to visually guide their direction to swim in a straight line may be a suitable sport-specific test of functional vision. However, this test may be less appropriate for classification purposes because a VI athlete’s ability to swim in a straight line may improve as a result of training; for instance, some VI athletes have been reported to adapt their stroke to feel for the lane ropes [14], or even to learn to use the feel of waves reflecting from the ropes to better direct themselves. Subsequently, tests of functional vision that directly evaluate the ability to perform a visually-dependent task are likely to be sensitive to improvements as a result of training, and would therefore unfairly penalise those who have adapted to their impairment.

Whereas most tests of functional vision are highly task-specific and therefore differ according to the task in which performance is being evaluated, tests of visual function are generic tests that are similar irrespective of the task. For VI classification, generic tests are likely to be the most suitable tests of vision, as long as impairment to that visual function is known to have a direct impact on performance in the sport. Research is required to establish the relationship between performance on those tests of visual function and performance in the sport of interest, or in a component of that sport that influences overall performance (for examples, see [15–22]). Consequently, a specific level of impairment in visual function could result in very different conclusions about the functional vision of the athlete, depending on the visual demands of the particular sport.

Generic tests of visual function offer a number of advantages that make them suitable for classification. First and foremost, performance is unlikely to change on tests of visual function as a consequence of training, and should therefore only change if the underlying medical condition causing the impairment has changed. Although tests used for classification should not be chosen on the basis of practical reasons, tests of visual function help to streamline classification because a single test of visual function could be used during classification for a range of different VI sports, and those tests are more likely to be familiar to the clinicians who conduct classification. In some cases, it might still be desirable to use a sport-specific rather than generic test of visual function; for instance, classification could be streamlined if there was to be a single sport-specific test of visual function that could replace multiple individual tests of impairment.

### The Incorporation of Additional Tests for VI Classification

VI is presently classified only on the basis of an athlete’s performance on tests of visual acuity and/or visual field, leading to concerns that classification may fail to accurately capture the full impact of VI on sport performance [9]. This is of particular concern because there is likely to be significant variation in the visual function of VI athletes who might have the same visual acuity, but which is caused by a range of different medical conditions. For instance, there may be significant variation in an athlete's ability to perceive contrast, depth, movement, and to see in the presence of bright light, depending on the medical condition causing their impairment. An impairment to any one of those additional measures of visual function could have a significant impact on performance in, for example, alpine skiing, yet those aspects of vision are not presently tested during classification. Instead, the present system would evaluate the vision of athletes as being identical if there is no difference in the extent of the impairment to their visual acuity (or visual field). Moreover, it is entirely possible that other potential athletes are excluded because they perform well on tests of visual acuity and field, yet still have an impairment in other aspects of visual function that decrease their skiing performance. Those athletes would be deemed not eligible (NE) to compete in VI competition. Accordingly, there has been a call from athletes and others in VI sport to consider the inclusion of additional tests of vision during classification [9].

An evidence-based system of classification is necessarily sport-specific and will therefore most probably lead to the adoption of a unique selection of tests of visual function to account for the particular visual demands of each sport. There is a range of different tests that could be considered for inclusion in classification (e.g. acuity, fields, contrast, colour), and therefore, for any given sport, researchers must first narrow down that candidate list by determining the aspects of visual function that might be related to performance in that sport. Those aspects of vision could be established by (1) a considered breakdown of the subtasks performed within the sport and the nature of the visual information likely to be required to perform those tasks (e.g. Erickson [23]); (2) determining the types of impairment underrepresented in that sport by comparing the impairments common to athletes who play in that sport with those in the overall VI population; and/or (3) an expert consultation process (e.g. via a Delphi review [8, 9]) whereby VI athletes and their coaches are asked about the aspects of the sport that athletes find challenging as a result of VI (athletes who acquired their impairment after starting to play the sport can be particularly helpful in this regard).

Once the candidate tests of visual function have been established, research can be performed to provide evidence to demonstrate which of those tests are related to performance. Specifically, if impairment to the visual function is associated with a significant decrease in performance in that sport, then the test should be considered for inclusion in classification. However, for the test to be actually incorporated into the classification procedure, the addition of the new test should improve the ability of the classification system to minimise the impact of impairment on the outcome of competition. That is to say, when the test is included, the outcome of competition should be less related to the level of impairment of the athletes competing than what would be possible if the test were not included. It could be that only a single measure, several measures, or a weighted combination of measures may be required [8, 24].

Although an evidence-based system could conceivably lead to a different selection of vision tests being adopted for classification in each VI sport, many of the visual functions being tested are likely to be common across the sports. In order to streamline classification, the ideal scenario would be to identify one particular test for each of the visual functions. If each sport were to use a different test of the same function (e.g. contrast sensitivity), then classifiers would be required to become familiar with the range of different tests, or specific classifiers would be required for each sport. In order to deal with this issue, an expert meeting of vision specialists has been convened to recommend the ‘ideal’ classification test for each of the visual functions most likely to be related to performance in sport. Those performing classification research are encouraged to choose from those tests wherever possible to aid in streamlining classification.^1^

### Testing the ‘Best’ Eye or Both Eyes Together

Classification has historically been performed on the basis of the vision test results obtained when testing the better performing of the two eyes, although this approach has recently been called into question [9]. When in competition, in most cases athletes use both eyes simultaneously, with aiming sports such as shooting and archery being two exceptions. Therefore, the level of vision established during classification may fail to accurately capture that used during competition [12]. When compared with testing the best eye alone, the vision of some athletes may improve if tested with both eyes together (combining the information from the two eyes), while conceivably for some athletes vision could be worse when using two eyes (e.g. in some forms of nystagmus). Therefore, classification should not necessarily rely on the test results for the best eye only, but instead, for some sports, consider the use of both eyes *together*. The choice to use the best or both eyes can be made in one of two ways. The first would rely on a simple evaluation of what the habitual situation is likely to be when competing in that sport. That is, if the majority of athletes would typically use both eyes in that sport, then classification should be performed based on the results obtained when testing both eyes together. Research can then establish the relationship between impairment and performance when vision is tested using both eyes. The second option would be to test vision during research when using both eyes together and using the best eye only (or when using a score combining the test results using each eye individually and both together; see Colenbrander [25]); the test results that best predict variations in performance would provide an evidence-based decision as to whether to classify on the basis of one or both eyes.

### Testing ‘Best Corrected’ Vision

The athlete evaluation performed during classification is always performed with the athlete wearing their best possible optical correction (glasses or contact lenses; e.g. IPC [26]), even if the athlete does not wear that correction during competition. The requirement for athletes to wear their best optical correction when tested is in place because it establishes the best possible vision of the athlete; if it were not in place, many people with fully correctable short- or long-sightedness could simply remove their glasses/contact lenses and qualify to compete in para-sport. The requirement for best correction during classification does remain somewhat controversial though [9] because it is not always practical for a VI athlete to wear the correction during competition (e.g. to wear glasses while competing in judo or when swimming). Consequently, some athletes compete with a level of visual function that is markedly worse than that which was measured during classification.

Although athletes must wear their best correction during classification, this may not necessarily be the case when taking part in classification research. A primary goal of classification research is to establish the relationship between vision and performance during competition. Consequently, this relationship should be established on the basis of the athlete’s level of vision during competition. That is to say, if the athlete were to compete without any correction in place, then when tested for the purposes of classification research, the athlete should be tested without their correction. Failure to do so may lead to an overestimation of the impact of VI on performance. For instance, take an athlete whose impairment is only mild when corrected, yet is more severe when uncorrected. If the athlete was to compete uncorrected, then the impact on performance may be greater than what it would have been if corrected. However, if the athlete’s impairment had been evaluated for research purposes when corrected, then it would appear as though this mild impairment had a stronger impact on performance than it really did. Therefore, research should evaluate the athlete’s habitual vision during competition to develop a more accurate understanding of the relationship between impairment and on-field performance.

### Ambient Lighting During Classification

Classification for VI sports takes place indoors, often in a clinic or hospital, meaning that the lighting conditions may differ markedly from those experienced during competition. For sports played outdoors, the lighting is typically much brighter during competition, and can vary enormously, when compared to the controlled conditions experienced during classification. Even for sports played indoors, athletes can be required to adapt to large differences in lighting, for instance in swimming pools with large windows, or when bright lights are used for broadcast television. This would not represent a problem if changes in ambient lighting were to impact the vision of all athletes similarly. However, an athlete’s ability to adapt to changes in lighting can be impacted by some medical conditions more than others (e.g. Cornelissen et al. [27]). For example, albinism typically impacts vision in bright lighting, while retinitis pigmentosa and some cataracts can selectively reduce vision in dull conditions. As a result, the test results obtained indoors could be a poor predictor of performance in outdoor conditions and in some indoor scenarios.

The decision whether to account for the impact of lighting on vision during classification should be sport-specific. Some support has been expressed for classification to take place outdoors for athletes who take part in outdoor sports [9]; however, such an approach is likely to be indefensible because outdoor lighting levels can vary drastically, and therefore the outcome of classification would fluctuate depending on the lighting. This would lead to some athletes being classified in bright conditions, while others when it is dull, and might encourage some athletes to selectively seek opportunities for classification in conditions that suit them, resulting not only in an approach that is inequitable, but one that would ultimately be open to legal challenge. Instead, the best approach is likely to be for all sports to continue to classify indoors, but with modifications to the procedure to account for the impact of lighting on vision. Unfortunately, it is not practical indoors to fully simulate the level of lighting experienced outside. Instead, classification should attempt to account for the ability to adapt to different lighting conditions [28]. Performance on tests of visual function such as visual acuity and/or contrast sensitivity can be assessed in normal indoor conditions, in addition to when performed in the presence of glare (e.g. using the brightness acuity tester [29]), and when it is dark (e.g. using low-light transmission lenses [30]). Moreover, the speed of adaptation and/or recovery can also be tested in sports where athletes may be required to adapt to rapid changes in lighting, for instance when skiing through a forest or accounting for shadows on a playing field.

### The Relative Impact of Congenital and Acquired VI

Para-athletes often have an impairment not only in their ability to perform during competition, but also sometimes in their ability to benefit from training. Successful training outcomes can rely on: access to facilities, equipment, and coaching; having the available time necessary for training; and on possessing the capability to actually travel to a training venue. Conceivably, those with greater impairment may find it more challenging to take part in training. As outlined in the 2011 Position Stand, classification should not account for the impact of impairment on the ability to train, and should instead focus solely on the impact on performance during competition [4]. Indeed, an athlete who has maximised their training opportunities should not be penalised by being placed into a class that requires them to compete against others with less impairment but who trains less. Nonetheless, in VI sport, there is some concern that classification should, in some cases, take into account the impact of impairment on training, not to account for the ability of athletes to *access* training, but rather because some athletes have a fundamental impairment in their ability to acquire skill during training [9]. In particular, the age at which the VI was acquired could impair an athlete’s ability to acquire both their basic movement skills and their more advanced sport-specific skill during training.

Vision can play a decisive role in the development of motor skill, therefore a person with any degree of congenital VI could experience considerable difficulty in their ability to acquire fundamental motor skills [31]. Children learn to model their movements on those of others, with this process of observational learning [32] providing the cornerstone for much of our early motor development. Similarly, success in many sports requires an athlete to learn novel complex movements (e.g. swimming and judo), therefore the skill development of an athlete is likely to be slower if they cannot learn those movements with the benefit of vision. Consequently, an athlete with congenital VI may possess a fundamental limitation in their ability to acquire some motor skills [33]. The crucial implication is that an athlete with congenital VI might not be able to compete equitably against other athletes who acquired their VI later in life, even if they possess an identical level of vision when measured using standard tests of visual function. This has led some to call for classification to take into account the age or developmental stage at which the impairment was acquired so that those with congenital impairment can compete more equitably [9].

Caution is warranted though when assuming that those with congenital impairment are always at a disadvantage because there may be some situations in which there is no disadvantage. For instance, the role of vision may be minimal when learning relatively simple sport-specific movements such as those required for cycling and rowing. In those sports, the age of impairment may have little or no effect on the ability to acquire the movement necessary to perform the skill. There may even be certain cases in which a congenital impairment provides an *advantage*; for instance, a congenital impairment could provide an athlete with the opportunity to acquire more hours of deliberate practice in the VI form of the sport [34]. Moreover, a congenital VI can sometimes lead to sensory substitution, whereby there are improvements in the responsiveness to other sensory information (e.g. hearing and touch [35]). Although speculative, this compensation could lead to specific advantages in some scenarios, for example in VI shooting where the sport is adapted so that athletes can use the pitch of a sound to align their gun towards the target. It is possible that those with more acute sensitivity to auditory stimuli could have an advantage in auditory guidance and therefore in overall shooting performance [21, 22].

Given the view outlined in the 2011 Position Stand that classification should not take into account the impact of impairment on the ability to train [4], there is considerable uncertainty whether the age at which VI is acquired can or should be accounted for during classification. Moreover, there is some concern that to do so would overcomplicate classification [9]. This position may change if there was to be clear evidence that the impairment–performance relationship is moderated by the age at which an impairment is acquired. Those conducting classification research are encouraged during their research to collect background information that maps the development of the athlete’s impairment alongside their development of skill in the sport, ideally employing a developmental history of practice volume in the sport [36, 37], along with a survey of the age the impairment was acquired and the rate at which it progressed. As a result, research can establish whether the age of acquisition does influence performance, and can help lead to an evidence-based decision about whether the age of acquisition should be factored into classification for that sport in the future.

## Models for VI Classification Research

A variety of approaches can be adopted to establish the relationship between impairment and performance during VI classification research. A truly evidence-based system of classification is likely to require a combination of those approaches. For instance, because of the adaptations used in VI sports, some approaches are more suitable for establishing the minimum impairment criteria, while others are more suitable for determining sport classes. Here we describe what are likely to be the three most important models for classification research, and discuss how each can contribute to the development of evidence-based classification (see Table 1 for an overview).

**Table 1:** Outline of three research models that can be used for VI classification research

Research model	Suitability for the development of:			Advantages (+)/disadvantages (−)
	Minimum impairment criteria	Sport-class criteria	Tests to be used during classification	
Impairment–performance relationship in VI athletes (correlation model)	~ The existing MIC may limit the ability to find skilled individuals with less severe impairments	✔ Can determine the impact of the complete range of VI on performance in the adapted form of the sport	✔ Can determine which tests are related to performance and therefore are suitable for classification	+ Can test participants with VI (i.e., real medical conditions) − The current classification system affects the characteristics of the participants − Other factors (e.g. training volume, adaptation, talent) also influence performance and should be accounted for
Simulation of vision impairment in skilled fully-sighted athletes (simulation model)	✔ A range of VI severities both below and above the current MIC can be simulated to determine the least severe impairment that starts to impact performance	~ Sport-class criteria should be set based on the impact of the impairment on performance in the adapted form of the sport	✔ Can determine whether certain aspects of visual function impact performance. This can be used to decide which aspects of visual function to assess during classification	+ Can measure the direct effect of impairment on performance, without the effect of long-term adaptation through training + Possible to test outside the range of impairment severities currently eligible − Not all impairments can be simulated − Not all VI sports have sighted equivalents
Establishing the visual information relied on by skilled fully-sighted athletes (component-analysis model)	✔ The visual information relied on by skilled athletes could be translated into the VI severity that limits the ability to detect this information	~ Sport-class criteria should be set based on the impact of the impairment on performance in the adapted form of the sport	✔ New sport-specific tests can be designed that assess whether the athlete can detect the visual information that skilled athletes rely on	+ No bias from other (indirect) effects of vision impairment confounding the impairment–performance relationship − Not all VI sports have sighted equivalents − The visual information used by able-sighted athletes might not match that used by those with VI

*VI* vision impairment, *MIC* minimum impairment criterion

### Establishing the Impairment–Performance Relationship in Athletes with VI

The impairment–performance relationship can be established by measuring the vision of athletes who already participate in para-sport, and correlating the outcomes with measures of the performance of those athletes. Therefore, two key decisions need to be made using this ‘correlation’ approach: (1) which aspects of visual function should be measured (see Sect. 3.2); and (2) how best to measure performance in that sport. The evaluation of sport performance could involve measuring overall performance, particularly in sports where performance is measured objectively (e.g. race-time in swimming or throw-distance in javelin). Otherwise, measures could evaluate important determinants of performance when the measurement of overall performance is more complex (e.g. the ability to kick towards a target in football or to intercept a ball in goalball). It is wise to consult experts in the sport to establish the aspects of performance likely to be impacted by VI, and to determine how they are best measured. Performance analysts who scrutinise and measure performance can also be particularly useful in providing advice.

Having measured the visual function and performance of a sufficiently large group of athletes, the outcomes can be correlated and subsequently analysed to examine the nature of the impairment–performance relationship. The three main objectives here are to identify (1) those aspects of vision that best predict sport performance; (2) the most appropriate number of sport classes; and (3) the most appropriate sport-class boundaries. To address the first objective, traditional statistical techniques such as a correlation or multiple linear regression would be appropriate. However, for the purpose of determining the most appropriate number of classes, as well as the criteria for separating those classes, a number of contemporary approaches, such as cluster or decision-tree analyses, are more likely to be suitable (for more detail, see Tweedy et al. [8]). These approaches can help to determine the measure of visual function, or most likely the combination of measures, that should be used to allocate a sport class during evidence-based classification.

Because this correlation approach assesses athletes who already compete in VI sport, it will most likely involve an examination of performance in the adapted rather than unadapted form of the sport. This makes the approach very useful when establishing the number of sport classes and the borders between them, but may be less suitable for establishing the minimum impairment criteria. However, there are still some scenarios in which this approach is suitable for establishing the MIC; in particular, (i) when the VI form of the sport is identical to the unadapted version; or (ii) when data for sport performance can be collected for those VI athletes competing in the unadapted rather than adapted form of the sport (e.g. by a specially convened competition for the purposes of research).

The principal advantage of this research design is that it can examine the impairment–performance relationship in athletes with impairment when competing in VI sports. Instead of attempting to make inferences on the basis of findings for athletes who do not have impairment, it tests athletes who have a variety of conditions that can impact all aspects of visual function. This provides a holistic impression of how impairments truly influence performance. However, this approach does have its drawbacks. Classification research should evaluate the impact of impairment on performance during competition [4]. Yet, the relationship between impairment and performance established when using this approach is likely to be influenced by other aspects of an athlete’s life that might indirectly influence performance, but are not easy to standardise in a population of VI athletes (e.g. age of impairment, volume and quality of training). In an attempt to overcome this limitation, additional athlete information should be gathered during research (see Sect. 3.6) to either include only those athletes who meet certain criteria or to control for those additional variables.

An important issue to consider when applying this model is that the present classification system will directly influence the characteristics of the athletes who participate in the research. First, people whose level of impairment is less than that required to meet the prevailing MIC may need to be recruited from the able-sighted equivalent of the sport. However, those individuals may be less likely to be actively competing if their impairment does decrease performance in the unadapted form of the sport. Moreover, they might not be accustomed to some of the adaptations, such as blindfolds and guides, that might be used in the adapted form of the sport. Second, there may be an underrepresentation of people with impairment who qualify for inclusion in the para-version of the sport but whose impairment under the existing classification system places them at a significant disadvantage when compared with others with less impairment within their sport class. Because of this disadvantage, those people may be less likely to be actively competing because, for example, they are not able to meet qualification standards to compete, or they decide to leave the sport. Coaches and administrators within the sports can be very useful in identifying athletes who fit those criteria. If those athletes are found at a local or regional rather than international level, the quantity and/or quality of their training experience may need to be taken into account. Ideally, improvements to a classification system should encourage wider participation and lead to a more representative sample of athletes with impairment taking part in the sport. It is therefore necessary to re-evaluate from time-to-time the impairment–performance relationship found for any newly developed classification system.

### Simulation of VI in Skilled Fully-Sighted Athletes

The relationship between vision and sport performance can be established by simulating VI in fully-sighted athletes and measuring changes in their sport performance. A number of different aspects of visual function can be manipulated in sighted athletes; for instance, changes in visual acuity, contrast sensitivity, and, in some cases, visual field. These manipulations can be achieved through the use of glasses (e.g. Applegate and Applegate [20], and Bulson et al. [18, 19]), contact lenses (e.g. Mann et al. [15, 16]), or by computer-based simulations, including gaze-contingent displays [38, 39]. However, it remains challenging, or indeed impossible, to manipulate some other types of visual function (e.g. eye movement disorders).

The simulation approach is most suitable for examining performance in the unadapted form of a sport because fully-sighted athletes would be untrained in the adapted format. As a result, the simulation approach holds promise for establishing the minimum impairment criteria, and may be less useful for establishing sport classes, again unless there are no or minimal differences between the unadapted and adapted forms of the sport. When simulating impairment, the skill level of the able-sighted athletes can be chosen so that it matches that of the VI athletes (e.g. matching race times in swimming) to maximise the relevance of the findings to the VI population.

A particular advantage of the simulation approach is that the impairment–performance relationship can be evaluated while controlling for the amount of time that an athlete has had available to adapt to their impairment. When using the *correlation* approach to test para-athletes, it is not always easy to differentiate whether the better-performing athletes are superior because they have less impairment, or rather because they have had more and/or better-quality practice, and are therefore more adapted to their impairment than less-trained athletes. Therefore, a research approach is desirable that can establish the ‘raw’ impact of the impairment on performance (i.e. while controlling for adaptation). Note though that when establishing the MIC using the simulation approach, it is important that participants without impairment have had at least some time to adapt to the newly imposed impairment. Existing studies that simulate impairment generally provide athletes with 5–15 min to familiarise themselves with any acute effects of the simulated impairment before testing performance [15, 16]. Given that a longer period of adaptation is likely to provide athletes with more time to develop strategies to adapt to or overcome the newly imposed activity limitation, then it stands to reason that insufficiently short adaptation times will result in an MIC that requires less impairment than that which might genuinely limit performance over the long-term. That is, an insufficiently short period of adaptation would overestimate the impact of VI on performance, ultimately leading to the inclusion of athletes whose impairment results in only a transient impact on sport performance. A longer period of adaptation would help to overcome this concern, and is desirable for setting an MIC that truly reflects the long-term impact of impairment on performance.

Of course there are limitations to the practicality of the simulation approach. First, some sports might have been created specifically for VI athletes and would therefore not have a sighted equivalent (e.g. goalball). In those cases, it would be challenging to find able-sighted athletes who are skilled in the sport. One possible solution is to evaluate the impact of simulated impairment on performance in a sporting task that is very close to that performed in the VI sport. For example, to test changes in performance in goalball (where competitors attempt to roll a ball into their opponents’ goal), the interceptive ability of skilled team handball players could be tested as a representative task. A second potential limitation is that athletes who do not have VI have learned to play the sport with the benefit of vision, and it could be that some elements of their performance are so well-learned that their skills are more ‘automatic’ and rely on different sources of information to that of lesser-skilled athletes [40, 41] or athletes with VI. Third, it is not possible to simulate an impairment to all the types of visual function that may be relevant to sport performance. Finally, some VI athletes prefer that classification rules should be created on the basis of the performance of athletes who have VI, rather than on the performance of those who do not [9]. In particular, there is apprehension that simulations in athletes without VI fail to capture the overall impact of the impairment on the athlete’s ability to train and to acquire fundamental and sport-specific motor skills. Moreover, caution is of course required when temporarily impairing the vision of these athletes, i.e. test procedures must meet the requirements of the local Ethics Committee; athletes should be prepared for the physical and psychological reactions they may experience during testing; and an appropriate debrief is advised to avoid athletes being left with a negative perception about what it is like to live with VI.

### Establishing the Visual Information Relied on by Skilled Able-Sighted Athletes

This final component-analysis approach seeks to identify the visual information that skilled able-sighted athletes rely on for best performance in a given sport, and then determines the level of vision necessary to use that information. This approach is necessarily sport-specific because it requires a considered analysis of the performance-determining components of each sport, and what might be the visual information relied on when performing those tasks. Subsequently, the visual information can be manipulated in an attempt to demonstrate if and when it would be used to facilitate performance. For instance, the turn is a crucial component of most swimming races and it is likely that swimmers rely on the position of the ‘T’ on the bottom of the pool to initiate their turn. By examining changes in the turn in response to manipulations in the position of the ‘T’, it is possible to show when in the swim that information is used, and therefore the quality of vision that would be required to see that information [42] (for examples in long-jump, see also Bradshaw et al. [43, 44] and Panteli et al. [45]). Once the visual information relied on by skilled fully-sighted athletes is known, the level of vision required to use that information can be established, and thereby the level of VI likely to limit performance.

An advantage of this model is that it can lead to the design of sport-specific tests of visual function that hold promise as tests of classification. These tests can simulate the demands required during competition and could eliminate the need for separate tests that individually evaluate different aspects of visual function. For example in swimming, it could be that a test that discriminates whether an athlete is able to identify the direction of a ‘T’ similar to that seen in the pool could replace the need for individual tests of acuity, contrast, and colour. A benefit of this type of sport-specific testing is that it may require fewer tests for classification, although this does need to be weighed against the desire for as few classification tests across sports as is possible and the need to ensure that test performance does not improve in response to training in the sport [4].

Because this approach considers performance in the unadapted format of the sport, it too holds many of the advantages and drawbacks seen in the previous section for the simulation approach, i.e. it is likely to be most beneficial when establishing the MIC; it does offer the benefit of being able to reduce bias from other factors that indirectly influence performance (e.g. training volume); and it does rely on the assumption that the visual information relied on by VI athletes would match that relied on by athletes without VI.

## Conclusions

The vision of the IPC is to “enable para-athletes to achieve sporting excellence and inspire and excite the world” [46]. An appropriate system of classification is a significant requirement for the legitimacy of para-sport, and an evidence-based form of classification will help to ensure legitimacy. For athletes with VI, the progress towards evidence-based classification has, to date, been slow. The aim of this joint Position Stand is to provide guidance for how evidence-based classification research should be conducted in VI sports. The differentiation between the adapted and unadapted form of a sport holds significant implications for how evidence-based standards are developed for both the minimum impairment criteria and for the design of sport classes. Moreover, the outcomes of classification research may inform recommendations for the design of sport rules; for instance, the need to reconsider which athletes should use blindfolds during competition. The appropriateness of a sport-specific approach to classification also extends to the conditions in which the athlete evaluation takes place, in particular ensuring that appropriate sport-specific decisions are made about the addition of new tests and the lighting used when performing those tests. Finally, there is no single approach to take when performing research to establish an evidence-based system of classification. A robust system is likely to require a combination of approaches.

There are a number of future challenges that need to be overcome to meet the needs of a legitimate system of classification for athletes with VI. New tests of visual function are required to better account for the impact of VI on sport performance, with those tests needing to be suitable for people with VI and to be representative of aspects of visual function that influence sport performance. An additional challenge is to establish whether a single aspect of vision is capable of describing the impact of impairment on sport performance, or rather whether a particular combination of visual functions should be used. The relationship between impairment and performance is likely to be confounded by other factors such as practice volume and age, therefore these factors should also be measured and controlled wherever possible. When these particular challenges are met and new systems of classification are developed, a key task will be to ensure that appropriate athlete education is in place during implementation. VI athletes are very accustomed to their present system and some may be resistant to change, particularly those who benefit from the current system. Education is required to ensure that all athletes are aware of the purpose of sport-specific classification and the long-term benefits to all athletes. Even the best evidence-based system of classification will not be successful if it is not accepted by the athletes for whom the new system is designed to help.

## Footnotes

1 At the time of writing, the details of these tests that were chosen by the expert panel were not yet published. For further information, please contact the corresponding author.
